# Methodological challenges in European ethics approvals for a genetic epidemiology study in critically ill patients: the GenOSept experience

**DOI:** 10.1186/s12910-019-0370-1

**Published:** 2019-05-07

**Authors:** Ascanio Tridente, Paul A. H. Holloway, Paula Hutton, Anthony C. Gordon, Gary H. Mills, Geraldine M. Clarke, Jean-Daniel Chiche, Frank Stuber, Christopher Garrard, Charles Hinds, Julian Bion, The GenOSept National Coordinators, European Society of Intensive Care Medicine, Jordi Rello, Gonzalo Sirgo, Paolo Cotogni, Apostolos Armaganidis, Thomas Ryan, Frank Bloos, Ivan Novak, Vladimir Gasparovic, Ilona Bobek, Adam Mikstacki, Jan Hazelzet, Yoram Weiss, Antonio Carneiro, Silver Sarapuu, Pierre Damas, Maja Surbatovic, Vladimir Sramek

**Affiliations:** 1 Whiston Hospital, Prescot, Merseyside and Department of Molecular and Clinical Pharmacology, University of Liverpool, Liverpool, UK; 2 Imperial College, London, UK; 3 Intensive Care Unit, John Radcliffe Hospital, Oxford, UK; 4 University of Sheffield, Sheffield, UK; 5 The Health Foundation, London, UK; 6 Hospital Cochin, Paris, France; 7 Department of Anaesthesiology and Pain Medicine, Bern University Hospital and University of Bern, Bern, Switzerland; 8 Barts and the London Queen Mary School of Medicine, London, UK; 9 Institute of Clinical Sciences, University of Birmingham, Birmingham, UK; 10 GenOSept National Coordinators, European Society of Intensive Care Medicine (ESICM), Rue Belliard, 19, B-1040 Brussels, Belgium

**Keywords:** GenOSept, Genetic epidemiology, Research ethics, Decision-making, Human genetics, Intensive care

## Abstract

**Background:**

During the set-up phase of an international study of genetic influences on outcomes from sepsis, we aimed to characterise potential differences in ethics approval processes and outcomes in participating European countries.

**Methods:**

Between 2005 and 2007 of the FP6-funded international Genetics Of Sepsis and Septic Shock (GenOSept) project, we asked national coordinators to complete a structured survey of research ethic committee (REC) approval structures and processes in their countries, and linked these data to outcomes. Survey findings were reconfirmed or modified in 2017.

**Results:**

Eighteen countries participated in the study, recruiting 2257 patients from 160 ICUs. National practices differed widely in terms of composition of RECs, procedures and duration of the ethics approval process. Eight (44.4%) countries used a single centralised process for approval, seven (38.9%) required approval by an ethics committee in each participating hospital, and three (16.7%) required both. Outcomes of the application process differed widely between countries because of differences in national legislation, and differed within countries because of interpretation of the ethics of conducting research in patients lacking capacity. The RECs in four countries had no lay representation. The median time from submission to final decision was 1.5 (interquartile range 1–7) months; in nine (50%) approval was received within 1 month; six took over 6 months, and in one 24 months; had all countries been able to match the most efficient approvals processes, an additional 74 months of country or institution-level recruitment would have been available. In three countries, rejection of the application by some local RECs resulted in loss of centres; and one country rejected the application outright.

**Conclusions:**

The potential benefits of the single application portal offered by the European Clinical Trials Regulation will not be realised without harmonisation of research ethics committee practices as well as national legislation.

**Electronic supplementary material:**

The online version of this article (10.1186/s12910-019-0370-1) contains supplementary material, which is available to authorized users.

## Key messages

- The survey highlights the diversity in ethics assessment and approval procedures at national and local level across the EU for research involving genetic material and patients lacking capacity
- The improvements introduced by the European Clinical Trials Regulation to limit the adverse consequences of such variation are unlikely to be realised if current national variations in interpretation of research ethics guidance persist
- To improve the coherence and integration of ethics committees decision-making additional measures may be required to ensure consistent interpretation of national law across Europe

## Background

Sepsis has been described as “one of the oldest and most elusive syndromes in medicine” [1]. Sepsis is a condition with high mortality risk. Many factors, such as genetics, age, gender, ethnicity, comorbid conditions, number of dysfunctional organs and temporal trends in markers of acute physiological derangement have been associated with sepsis outcomes [2–9].

The GenOSept project was conceived by the European Critical Care Research Network of the European Society for Intensive Care Medicine (ESICM) to investigate the potential impact of genetic variation on the host response and outcomes in sepsis. It was part-funded in 2004 for 4 years by the European Union 6th Framework Programme (https://www.esicm.org/research/trials/endorsed-trials/completed-projects-supported/). The collaboration has continued since through additional specific project funding. The aims of the project were to identify possible genetic determinants of outcome from sepsis in an international cohort of critically ill patients, and to build an intensive care medicine genetics collaboration between clinicians and scientists across Europe. GenOSept was launched in January 2005, with 18 countries and 160 intensive care units (ICUs) participating. Three genome centres (in Bonn, Paris and Oxford) supported the project. A total of 2257 evaluable patients were recruited between May 2006 and December 2008, providing important insights into the epidemiology and genetics of sepsis in Europe [10–16]. Collaborative analytical work continues using the samples which are stored in biobank facilities at the Wellcome Trust Centre for Human Genetics in Oxford.

### European research: regulatory framework

A key component of the set-up phase of GenOSept was to determine the approval processes and outcomes of local and national ethics committees presented with a common protocol for genetic analysis in critically ill patients, many of whom would lack capacity. There are several European-level regulations of relevance to genetics research (Table 1). At the time GenOSept was conceived in 2004, clinical trials performed in countries within the European Union (EU) were required to adhere to the requirements of Good Clinical Practice described in the European Clinical Trials Directive 2001/20/EC (http://ec.europa.eu/health/human-use/clinical-trials/directive/index_en.htm), which was issued in April 2001 and transposed into the national laws of each EU member state by 2004. Individual institutions within EU countries are not permitted to introduce different research ethics legislation and are expected to adopt the principles of the EU Directive, by a process of “transposition”. Such process requires the EU member states to enforce the directive by passing appropriate legislative implementation measures. Individual nations within the EU may also adopt their own national ethical guidance, which may (or may not) be accompanied by a national review process (or a local review process based on national guidance).

**Table 1:** European regulations, position statements and advisory bodies affecting clinical research

Document	Source	Impact
Helsinki Declaration and the Universal Declaration on the human genome and human rights adopted by UNESCO (1997)	http://www.unesco.org/new/en/social-and-human-sciences/themes/bioethics/human-genome-and-human-rights/	Need for legal representative or deferred consent, in the event of incapacity; Genetic counselling; Research should ‘*contribute to the health benefit of other persons in the same age category or with the same genetic condition...*’
International Declaration on Human Genetic Data (2003)	http://www.unesco.org/new/en/social-and-human-sciences/themes/bioethics/human-genetic-data/	Recognition of ‘*special status*’ for human genetic data
The Charter of Fundamental Rights of the EU (2000)	http://www.europarl.europa.eu/charter/default_en.htm	Protection of personal data
European Directive on processing and free movement of personal data (Directive 95/46/EC)	http://eur-lex.europa.eu/LexUriServ/LexUriServ.do?uri=CELEX:31995L0046:en:HTML	Protection for individuals about the processing and free movement of personal data
European Group on Ethics in Science and New Technologies	https://ec.europa.eu/research/ege/index.cfm	An advisory body to the European Commission on ethical aspects of science and new technologies
European Clinical Trials Directive 2001/20/EC	http://www.eortc.be/Services/Doc/clinical-EU-directive-04-April-01.pdf	Requirement for prior informed consent from legal representative made emergency research impossible; Semantic confusion of ‘therapeutic’ and ‘non-therapeutic’ research
The International Conference on Harmonisation Guidance on Good Clinical Practice (Topic E6) (CPMP/ICH/135/95)	http://www.ema.europa.eu/docs/en_GB/document_library/Scientific_guideline/2009/09/WC500002874.pdf	Principles of good clinical practice in clinical trials research
The Good Clinical Practice Directive 2005/28/EC	http://eur-lex.europa.eu/LexUriServ/LexUriServ.do?uri=OJ:L:2005:091:0013:0019:en:PDF	Supplementing the Clinical Trials Directive
European Clinical Trials Regulation 2014	http://ec.europa.eu/health/human-use/clinical-trials/regulation/index_en.htm	Improving coordination of the application process for trials involving multiple countries, with creation of single EU entry point and trials databank

While the aims of the Directive were commendable in terms of attempting to harmonise research processes and safeguard persons enrolled in clinical trials, the guidance posed certain challenges which hampered the conduct of clinical research. The Directive failed adequately to recognise the special circumstances of research conducted in emergency care when patients may lack capacity and surrogates can be unavailable, and made no provision for ‘observational’ research lacking direct potential benefit to the participant. The application of the Directive was associated with increased economic, bureaucratic and administrative burdens and, especially in the case of multi-national studies, delays in the approval process, related to the fact that each member state’s research ethics apparatus could interpret the principles of the Directive in different ways. Given variations in national regulatory pathways, this resulted in research applications involving patients lacking capacity being rejected in some countries and approved in others, as we detail below.

Key ethical issues posed by the GenOSept project within this regulatory framework include the following:

I. The lack of capacity inherent in critical illness challenged the requirement to respect patient autonomy in obtaining consent.
II. The possibility of obtaining informed consent from surrogate decision-makers (‘legal representatives’) is much more difficult in the time-limited context of emergency care. This specific issue was mitigated by the possibility of obtaining blood for DNA testing for GenOSept at any time and patient data could be recorded retrospectively.
III. The European Clinical Trials Directive created a sematic confusion by referring to observational research as ‘non-therapeutic’, in the sense that such studies may have no direct benefit to the participants. However, they may benefit future patients or populations through enhanced scientific knowledge. The Directive did not acknowledge this important distinction.
IV. Public concerns about the security and privacy of genetic data [17] appeared to conflict with the study’s methodological requirement to transfer human genetic material across national borders for analysis in the genotyping centres.
V. The requirement to respect confidentiality of personal data required a study design which preserved individual de-identification while retaining the capacity to link genotypic with phenotypic data. Linkage might also be necessary in the event that a participant were retrospectively to request the results of analyses performed on their samples. Preserving individual de-identification, while also retaining capability for data reconciliation where required, poses questions around the true sense and limitations of anonymization of data, as informatics technique evolve and the potential for databases to be combined, integrated and cross-referenced increases.

To study the approaches to these issues taken by the research ethics committees, we therefore undertook an analysis of the processes and outcomes of ethics reviews across the European countries participating in GenOSept.

## Methods

A GenOSept project national coordinator was appointed in each country to identify and support ICUs to consent, recruit, and obtain a single set of blood samples from patients with severe sepsis or septic shock due to community acquired pneumonia (CAP), peritonitis, severe pancreatitis or meningococcal disease.

### Data protection

Individual de-identification was achieved while retaining the capability for data reconciliation, phenotypic integration and retrospective identification in the event of investigator enquiry, by using three coded numerical systems for clinical data, blood and DNA samples prior to the genomic analysis. A “linked anonymous” (de-identified) system involving a code specifying country ID (in letters e.g.UK), site ID (numerical) and patient ID (numerical) was used. This code was manually entered into the eCRF (electronic Case Report Form) and attached as a bar code to the blood samples and subsequently to the extracted DNA. Only the local clinician could link specific patients to their phenotypic data; the genome centres could only link the blood sample/DNA to the corresponding non-identified phenotypic data. The link between all three could only be made by an independent data Trustee, an academic lawyer from the UK with expertise in European legislation appointed by the project steering committee. It was stipulated that genetic information would not be made available to the patient.

### Ethics and consent

The protocol included information about current European research legislation and a detailed description of the directives or position statements available at the time. The information sheet and consent form included a description of the project in non-medical terms which national coordinators were responsible for translating into their respective languages.

Following submission of the project to research ethics committees (RECs) in each country, national coordinators were subsequently invited to provide details of the submission process and outcome at study set-up, and then again following establishment of the European Regulation. The survey aimed at evaluating the following aspects for each participating European Country:

- organisational arrangements (whether the approval procedure had been centralised at national level, or whether a local or regional process had to be followed)
- the number of intensive care units involved within each participating nation
- the form in which the application was made (whether via a web portal or in hard copy)
- the usual composition of the REC (detailing the number of lay members and those with medical or legal expertise)
- the month and year of submission for ethics approval and the duration of the process until approval
- whether approval was granted to all units within each nation, some units only, or whether refused
- the need for submission of further information to the REC
- whether national guidelines existed for the conduct of research in critically ill patients

The survey explored both whether national ethics guidance existed, and whether a national review and approval process was in place. Data on the baseline characteristic of the submission process and ethics approval was reported using descriptive statistics with absolute and percentage values, median and interquartile ranges, as applicable and relevant to each result. Where comparisons between groups of countries were required (with regards to the presence or absence of a centralised approval process, and whether national guidance was available or not), inferential statistics were conducted in the form of linear regression analyses.

## Results

Results are summarised in Table 2.

**Table 2:** Characteristics of the application process for Ethical approval across the various European countries involved in GenOSept

Country	National coordinator	No of ICUs	Is the ethics approval process centralized or local?		In what form is the ethics application made?		Which of the following are usual members of the ethics committees?			Outcome of GenOSept application process: range of centres approved			Month/Year ethics application submitted & decision received			Additional info / modification	National guidelines for Research in ICU pts
			National or Regional EC	Local EC	Paper application	Web-based Application	Lay	Medical	Legal	All units	Some units	Approval refused	Submitted	Approved or rejected	Interval (months)		
Austria	Novak	1	√	–	√	–	√	√	√	√	–	–	02/07	09/07	7	√	√^a^
Belgium	Damas	7	√	–	√	–	√	√	√	√	–	–	02/06	05/06	3	√	–
Czech Re	Sramek	8	√	√	√	–	√	√	√	√	–	–	07/05	07/05	< 1	√	–
Croatia	Gasparovic	2	–	√	√	–	–	√	–	√	–	–	11/07	3/08	4	–	–
Estonia	Sarapu	2	√	–	√	–	√	√	√	√	–	–	06/05	06/05	< 1	–	–
France	Chiche	8	√	–	√	–	√	√	–	√	–	–	10/05	10/06	12	√	√
Germany	Bloos	12	–	√	√	–	√	√	√	–	√	–	07/05	08/05	1	–	–
Greece	Armanagidis	2	–	√	√	–	–	√	–	√	–	–	04/06	04/06	< 1	–	–
Hungary	Bobek	10	√	–	√	–	–	√	–	√	–	–	10/05	09/07	24	√	–
Ireland	Ryan	7	–	√	√	–	√	√	√	√	–	–	03/06	10/06	7	–	–
Israel	Weiss	5	√	–	√	–	√	√	√	–	√	–	08/05	02/06	6	√	√
Italy	Cotogni	24	–	√	√	–	√	√	√	–	√	–	01/06	03/06	2	√	√
NL	Hazelzet	1	–	√	√	–	√	√	√	√	–	–	01/07	11/07	9	–	√
Poland	Mikstacki/Tamowicz	22	√	–	√	–	–	√	–	√	–	–	05/05	06/05	1	–	√
Portugal	Carneiro	0	√	√	√	–	√	√	√	–	–	√	08/05	08/06	< 1	–	–
Serbia	Surbatovic	2	–	√	√	–	√	√	√	√	–	–	07/06	07/06	< 1	–	√
Spain	Sirgo/Rello	19	√	√	√	–	√	√	√	√	–	–	07/05	08/05	1	–	√
UK	Hinds	28	√	–	–	√	√	√	√	√	–	–	03/06	04/06	1	–	√
Totals	18 countries	160	11	10	17	1	14	18	13	14	3	1	Median interval = 1.5 months			7	9

^a^Austria: the REC which received the application recommended inclusion only of those critically ill patients who had capacity to give consent

### Organisational setup, number of ICUs involved and format

Eighteen countries were willing to participate, incorporating 160 ICUs. The median number of ICUs involved in each country was 7 (interquartile range 2–12), the range being 1–28. Eight (44.4%) countries used a single centralised national or regional ethics committee for approval, seven (38.9%) required approval by an ethics committee in each participating hospital, and three (16.7%), Portugal, Spain and the Czech Republic, required submission to both a centralised and a local approval process. All countries used a paper-based application process except the UK, where a web-based system was in place. Results are summarised in Tables 2 and 3.

**Table 3:** Characteristics of the RECs across the various European countries involved in GenOSept

Characteristic	Median (IQR) or n (%) [Range]
No of ICUs per country	7 (2–15) [1–28]
Countries with centralized (national/regional) REC only	8 (44.4%)
Countries with local REC only	7 (38.9%)
Countries with both centralized and local REC	3 (16.7%)
Countries with paper application process	17 (94.4%)
Countries with online application process	1 (5.6%)
Medical members of REC	18 (100%)
Lay and Legal members of REC	13 (72.2%)
Lay but no Legal members of REC	1 (5.6%)
Approval at all units within country	14 (77.8%)
Approval at some units within country	3 (16.7%)
No approval at country	1 (5.6%)
Application process duration (months)	1.5 (1–7) [1–24]
Additional information or modification required	7 (38.9%)
Countries with National guidelines for research in critical care	9 (50%)

*IQR* inter-quartile range, *n* absolute number, *%* percentage

### Usual composition of RECs

Ethics committees included lay members in 14 (77.8%) countries and legal expertise in 13 (72.2%). In four (22.2%) countries (Croatia, Greece, Hungary and Poland) the ethics committees were exclusively composed of medical doctors, with no patient or public (lay) representation.

### Timing and duration of the approval process

Submissions for ethics approval were made, in the various countries, between May 2005 and November 2007. The median time from submission to final decision was 1.5 (interquartile range 1–7) months. In nine (50%) countries the application was approved within 1 month, while in six it took over 6 months; the duration was calculated as a “combined permissions process” (the times presented here were those including all regulatory and ethical steps between application submission and granting of permission to proceed). In Hungary the process of submission, initial rejection, and requests for clarifications and resubmission following appeal took more than 2 years before approval was granted, by which stage it was no longer possible to recruit centres.

Centralisation of approval did not confer greater efficiency. Median (Interquartile range) approval time in the countries with a centralised (national and/or regional) approval process was 1 (1–7) month, versus 2 (1–7) months in countries without such centralisation (linear regression analysis *p* = 0.57, r^2^ = 0.02).

If the nine countries with approval times of more than 1 month had been as efficient as those with approval times of 1 month or less, an additional 74 months of potential country or institution-wise recruitment would have been available in the first 2 years of the project.

### Approval outcomes

Ethics applications were approved for all participating ICUs in 14 countries. In three countries (Germany, Italy and Israel) the local ethics committees at some hospitals rejected the application, hence the study could only proceed in the remainder. In particular, in Italy approval was obtained for 14 (63.6%) of the 22 ICUs willing to participate; in Israel only for 5 (55.6%) out of 9 ICUs, while in the case of Germany the proportion was 12 (80%) out of 15 ICUs. This clearly indicates different interpretations of the same Directive within the same national regulatory environment. Portugal was unable to participate at all because one local committee and the national committee rejected the proposal.

Reasons for rejection of the application were diverse. They included disagreement about the acceptability of performing genetic research in incapacitated patients and taking blood from an unconscious patient (Italy), concerns about sending blood samples out of the country (Israel), and doubts about security of anonymity, validity of assent from relatives or legal representative, inclusion criteria and selection of genes to be studied, and concerns about unauthorised use of genetic data. In Portugal the application was rejected primarily because of concerns about the commercial use of human tissue and genetic data, as one of the scientific partners was SIRS-Lab, a university spin-off company; approval could not be obtained despite clear agreements about the use of intellectual property and the fact that the EU encouraged such partnerships.

### Need for submission of further information to the REC

In seven countries the applicants were required to submit additional information or modify the application. In Austria for example, the law required informed consent from the patient, thereby excluding patients without capacity; for this country the protocol was therefore modified to include only conscious patients capable of giving informed consent.

### Data access requests

No patient included in the study requested access to their information; hence the Data Trustee’s adjudication was not required at any time.

### National guidelines

Guidance at national level on the conduct of research in critically ill patients existed for 9 (50%) of the countries (Austria, France, Israel, Italy, the Netherlands, Poland, Serbia, Spain and the UK). Importantly, the fact that a country provides national guidance does not necessarily imply that there is a centralized review process to interpret such guidance. Indeed local institutions may interpret national guidelines in different ways. The existence of national guidelines was not associated with shorter time to approval, or with greater consistency in within-country decision making. Median (Interquartile range) approval time in the countries with existing national guidance was 1 (1–4) month, versus 2 (1–7) months in countries without guidance (linear regression analysis *p* = 0.91, r^2^ = 0.001).

## Discussion

We found substantial and persisting variations between EU member states in the organisation, structures, processes, efficiency, and decision-making of research ethics committees (RECs) in their assessments of an EU-funded observational study investigating the genetics of sepsis. In three countries decisions were inconsistent between individual centres, while one country did not allow any of its citizens to participate. The existence of national guidance was not always complemented by a centralized review process with uniform interpretation of such guidance. It seems paradoxical that, in two of the nine countries (Italy and Israel) where national guidance for ethics approval did exist, differential interpretation across the various local institutions led to approval being granted only for some of the centres willing to participate. In some centres delays in approvals and idiosyncratic requirements for protocol modifications hampered timely site initiation and patient accrual. If the nine countries with approval times of more than 1 month had been as efficient as those with approval times of 1 month or less, an additional 74 months of country or institution- level recruitment opportunities would have been realised in the first 2 years. Unnecessarily lengthy and laborious research ethics approval processes negatively impact on the perceived efficiency of the process, leading to increased dissatisfaction amongst academics [18]. The responses from some of the RECs involved in assessing the GenOSept application suggests unwarranted and potentially paternalistic exclusion of patients lacking capacity [19], the consequence being that critically ill patients may be excluded from benefiting from research participation.

The lack of standardized membership requirements for RECs which we have identified, and the recognised lack of a common ethics training curriculum, could also contribute to variation in practices and outcomes [20].

It is possible that RECs identified unique difficulties relating to cross-border genetics research which may have contributed to diversity in decision making. However, every REC received the same protocol for evaluation, and the critical care patients recruited to the various centres all met the same inclusion and exclusion criteria across the various EU countries. We therefore must conclude that the diversity in REC outcomes was attributable to non-clinical factors at an institutional, or national, level. Some of these factors must have been related to country-specific attitudes or legislation. Such diversity of decision-making between RECs is inconsistent with the principle of a harmonised approach to ethics across national borders. It is likely that attitudes amongst ethics committee members to genetics research in patients lacking capacity may have become modified in the years since the Human Genome project was completed.

Attempts to standardise the ethics of clinical research over many years [21, 22] have been hampered by the failure of the European Clinical Trials Directive 2001 [23] to accommodate the particular challenges of research in emergency settings [23–29], by widely differing interpretations of ethical principles by national or local RECs, as well as variations in approval processes [21–26]. The absence of harmonised processes and standardised interpretation delays studies, creates additional costs, and may prevent citizens from participating in research, while failing to provide added protection for participants [30–35].

The European Clinical Trials Regulation (http://ec.europa.eu/health/human-use/clinical-trials/regulation/index_en.htm) is a welcome attempt to resolve these difficulties (anticipated implementation in 2019). It requires that research applications are processed by one member state with the outcomes applying to all. This measure resembles the approach of the US National Institutes of Health, which have recently mandated the use of a single Institutional review board for multi-centre clinical studies. However, while the Regulation requires member states to cooperate in assessing a request for authorisation of a clinical trial, it does not include cooperation on matters ‘*of an intrinsically national nature, such as informed consent*’(paragraph 6), though it does state that ‘*ethics committees… should ensure the involvement of laypersons, in particular patients or patients’ organisations*’. It remains to be seen whether the requirement to process approvals through a single member state will solve the issue of wide variation in national research ethics processes and outcomes. It is evident that the current trials regulations, combined with the absence of standardised proceedures and training of RECs have increased the complexity and burdens of research governance, and have reduced the opportunity for participation in research, without evidence of benefit to participating subjects [36].

### Additional issues and challenges

An example of additional challenges faced by researchers in this area is provided by the UK. Here the Mental Capacity Act (2005) makes welcome provision for ‘non-interventional’ and emergency research; however, in the event of a consented person losing capacity during the research study, ‘advice’ must be obtained from the next of kin or equivalent consultee on whether the incapacitated person would have wished to continue with the study, even if the intervention has already occurred and that specific individual is now in the follow up phase [37]. If the consultee is of the view that the patient would not have wished to continue in the study, the patient must be withdrawn and the data destroyed or de-identified, unless the patient specifically consented to continue in the study in the event of loss of capacity.

A further example is the case of patients consenting to participate provided there was no risk that an organisation related to the government could access their data. In such a situation, medical, nursing and research staff would be allowed to use the data for the purposes of the study, but a government regulatory authority would not be allowed to review the notes or the data, even in the context of an inspection.

### Strengths and limitations

The GenOSept project began data collection over 10 years ago, and our survey demonstrates the challenges which continue to be faced by international researchers across member states involving genetic material and patients who lack capacity. Our findings show that efficient trans-European approval processes are possible. However, unexplained variation between some local and national ethics committees is having an undesirable effect on patient participation. While our survey did not allow for interaction with individual ethics committees to explore in greater detail the reasons for these variations in decision-making, we were able to use the information provided in the approval or rejection letters.

## Conclusions

Our study highlights the diversity and adverse consequences of variation in ethics assessment and approval procedures at national and local level across the EU for research involving genetic material and patients lacking capacity. The improvements introduced by the European Clinical Trials Regulation will not be realised if current national variations in interpretation of research ethics guidance persist. The invaluable service provided by these committees to patients and the research community may require targeted support to develop a common interpretation of European legislation and the moral assumptions underpinning research in critically ill patients lacking capacity.

## Additional file

Additional file 1: List of Contributing Centres and Investigators. (DOC 121 kb)

## Abbreviations

CAP: Community acquired pneumonia
eCRF: Electronic Case Report Form
ESICM: European Society for Intensive Care Medicine
EU: European Union
FP6 Sixth: Framework Programme (European Commission)
GenOSept: Genetics Of Sepsis and Septic Shock
ICU: Intensive care unit
MREC: Multicentre Research Ethics Committee
REC: Research Ethic Committee
