# Supplementary material for: Methodological challenges in European ethics approvals for a genetic epidemiology study in critically ill patients: the GenOSept experience
Source: BMC Med Ethics. 2019 May 7;20:30. doi: 10.1186/s12910-019-0370-1 (PMC6503539; doi:10.1186/s12910-019-0370-1)
Supplement: Supplementary file 1 — List of Contributing Centres and Investigators. (DOC 121 kb) [file 12910_2019_370_MOESM1_ESM.doc]

**GenOSept study**

**List of Contributing Centres and Investigators**

*Belgium*:

Intensive Care Unit, AZ-VUB university hospital, 101 Laarbeeklaan, Brussels; Intensive Care Unit, Chu Charleroi, 92 Boulevard Janson, Charleroi; Soins Intensifs, Clinique Saint Pierre, 9 Avenue Reine Fabiola, Ottignies; Intensive Care, Cliniques Universitaires Saint Luc (UCL), 10 Avenue Hippocrate, Brussels; Intensive Care, University hospital, 185 De Pintelaan, Gent; Soins Intensifs, Cliniques de l'europe - St Michel,150 Rue de Linthout, Brussels.

*Croatia*:

Medic, Emergency and Intensive Care Medicine/Internal Medicine, Clinical hospital Rebro, 12 Kispaticeva, ZagrebAnestesiology and ICU, Clinical Hospital Rebro, 12 Kispaticeva, Zagreb.

*Czech Republic*:

Anesteziologicko-reuscitacni klinika, Fakultní Nemocnice u Svaté Anny, 53 Pekařská, Brno; Anesteziologicko-resuscitacni oddeleni, Fakultni Nemocnice Brno, 20 Jihlavská, Brno-Bohunice; Klinika anestezie, resuscitace a intenzivni mediciny, Fakultni Nemocnice Hradec Kralove, 581 Sokolská, Hradec Kralove; Chirurgicka klinika, Fakultní Nemocnice s Poliklinikou Ostrava, 1790 listopadu, Ostrava-Poruba; Anesteziologicko-resuscitacni klinika, Fakultni Nemocnice Plzen, 80 Alej Svobody Plzen; Anestezie, resuscitace a intenzivni medicina, Masarykova Nemocnice, 3316/12A Sociální péče, Ústi Nad Labem; Anesteziologicko-resuscitacni oddeleni, Nemocnice Znojmo, 11 Janského, Znojmo; Anesteziologicko-resuscitacni oddeleni, Krajska Nemocnice Liberec, 10 Husova, Liberec.

*Estonia*:

General ICU, Tartu University Hospital, 1a L. Puusepa, Tartu; Pulmonary ICU, Tartu University Hospital,1a L. Puusepa, Tartu.

*Franc*e:

Service de Réanimation Médicale, Hopital Cochin, 27 rue du Fbg St Jacques, Paris; Service de Réanimation Médicale, HEGP, 20 rue Leblanc, Paris; Service de Réanimation Médicale, Hotel Dieu, 1 place du Parvis Notre Dame, Paris; Service de Réanimation Médicale, Saint Joseph, 185 rue Raymond Losserand, Paris; Service de Réanimation Médicale, Chru Angers, 4 rue Larrey, Angers; Service de Réanimation Médicale, Chu de Nice, Rte St Antoine Ginestière, Nice; Service de Réanimation Médicale, Chu Purpan, Chu Toulouse- Hôpital Purpan, Toulouse; Service de Réanimation Médicale, Ch Versailles, 177 rue de Versailles, Le Chesnay.

*Germany*:

Klinik für Herzchirurgie, Klinikum der Stadt Ludwigshafen am Rhein GGMBH, 79 Bremserstraße, Ludwigshafen; Klinik und Poliklinik für Anästhesiologie und Intensivmedizin, Klinikum Greifswald, 23b Friedrich-Loeffler-Straße, Greifswald; Klinik fur Anästhesiologie und operative Intensivmedizin, Klinikum Augsburg, 2 Stenglinstr., Augsburg; Klinik und Poliklinik für Anaesthesiologie und Intensivtherapie, Universitätsklinikum Dresden, 74 Fetscherstrasse, Dresden; Klinik für Anästhesiologie und Intensivtherapie, Klinikum der Friedrich Schiller Universität, 101 Erlanger Allee, Jena; Klinik für Anästhesie und Intensivmedizin, Westküstenklinikum Heide, 50 Esmarchstraße, Heide; Abt. fur Anästhesiologie und Intensivtherapie, Fachkrankenhaus Coswig - centre for pneumology and thoracic surgery, 21 Neucoswiger Str., Coswig; Klinikum der Medizinischen Fakultät der Martin Luther Universität Halle-Wittenberg, 40 Ernst-Grube-Str., Halle; Klinik für Intensivmedizin, University medical center Eppendorf, 52 Martinistr., Hamburg; Klinik und Poliklinik für Anästhesiologie und Operative Intensivmedizin (Turmgebäude 2OG Zimmer 221), Universitätsklinikum Bonn, 25 Sigmund-Freud-Str., Bonn; Internal Medicine, Universitätsklinikum Mainz, 1 Langenbeckstrasse, Mainz.

*Greece*:

Intensive Care, Sismanoglion general hospital, Marousi, Athens; Critical care, Attikon university hospital, 1 Rimini, Xaidari.

*Hungary*:

Surgery 1^St^, Semmelweis University, 78 Ulloi Ut, Budapest

*Eire*:

Intensive care unit, St James hospital, James Street, Dublin; Intensive care unit, Adelaide Meath and national children’s hospital, Tallaght, Dublin; Anaesthesia and Intensive care, National university hospital Galway, Newcastle Road, Galway; Anaesthesia & Intensive Care Medicine, James Connolly memorial hospital, Blanchardstown, Dublin; Department of Anaesthesia and Intensive Care Medicine, Cork university hospital, Wilton, Cork.

*Israel*:

Carmel medical center, Haifa; General Intensive care unit, Haemek medical center, Afula; Anaesthesiology and critical care medicine, Hadassah medical center, Kiryat Hadassah, P.O. Box 12000, Jerusalem

*Italy*:

Anestesiologia e Rianimazione 3, Ospedale S. Giovanni Battista – Molinette, 88 Corso Bramante, Torino; Anestesia e Rianimazione, Ospedale S.Giovanni Bosco, 3 Piazza Donatori del Sangue, Torino; Dr Rianimazione SOD 2, AOU Careggi, 85 Viale Morgagni, Firenze; Anestesia e Rianimazione, Ospedale Maggiore, 35 Via Francesco Sforza, Milano; Terapia Intensiva, Universita Degli Studi Milano Bicocca A.O. San Gerardo, 106 Via Donizetti, Monza; Anestesia e Rianimazione, Ospedale S.Orsola Malpighi, 9 Via Massarenti, Bologna; Anestesia e Rianimazione, Ospedale S.Giovanni Addolorata, 8 Via dell'Amba Aradam, Roma; Scienze Anestesiologische, Medicina Critica e Terapia del Dolore, Policlinico Umberto I, 155 Viale del Policlinico, Roma.

*Netherlands*:

Intensive care unit, Erasmus medical centre, 230 Gravendijkwal, Rotterdam.

*Poland*:

Anaesthesiology and Intensive Therapy, Medical university, 7 Debinki St, Gdansk; Klinika Anestezjologii i Intensywnej Terapii sp Centralny Szpital Kliniczny Sam; Military teaching hospital; Szpital Wojewodzki/regional hospital; University hospital n°2; Szpital Wojewodzki; University hospital of Bydgoszcz; Wroclaw medical University.

*Serbia*:

Military medical academy; Clinical center Kragujevac.

*Spain*:

Coordinating centre: Universitat Roira & Virgili / Hospital Universitari Joan XXIII de Tarragona, CIBERES. University hospital de Bellvitge; hospital Universitario Puerta del Mar; hospital Universitario de Gran Canaria; hospital de la Princesa; hospital Nostra Senyora de Meritxell; hospital de Mataro; hospital clinico San Carlos; hospital Universitari de Terragona Joan XXIII; hospital Sagunt; centro medico Delfos; hospital de Huesca; hospital general de Segovia; Basurto hospital; hospital Universitario Arnau de Vilanova; hospital general Yague; hospital Universitario Puerto Real; hospital Universitario de Girona; hospital General de Vic; Hospital Verge De La Cinta.

*United Kingdom*:

Aberdeen Royal Infirmary; Addenbrooke's Hospital; Barts and the London NHS trust; Broomfield hospital; Charing Cross Hospital; Chelsea and Westminster Hospital; Cheltenham general hospital; Colchester General Hospital; Freeman Hospital; Frimley Park hospital; Hammersmith hospital; Homerton University hospital; Hope hospital; Huddersfield royal infirmary; Hull royal infirmary; Ipswich hospital NHS trust; John Radcliffe hospital; Leeds general infirmary; Leicester royal infirmary; Manor hospital, Walsall; Norfolk & Norwich NHS trust; Queen Elizabeth hospital, King's Lynn ; Queen Elizabeth University hospital; Royal Berkshire Hospital; Royal Hallamshire hospital, Sheffield; Royal Preston hospital; Royal Sussex county hospital; Royal Victoria infirmary; Southend hospital NHS trust; st James University hospital; the Great Western hospital; the James Cook University hospital; The Whittington hospital; UCLH Middlesex hospital; University hospital Lewisham; University hospital of Wales; University hospital, Coventry; Worthing hospital; Wythenshawe Hospital

**GenOSept study**

**National Co-ordinators:**

| Austria | H Novak |
| --- | --- |
| Belgium | P Damas |
| Croatia | V Gasparovic |
| Czech Republic | V Sramek |
| Estonia | S Sarapuu |
| France | J-D Chiche |
| Germany | F Bloos |
| Greece | A Armagandis |
| Hungary | I Bobek |
| Ireland | T Ryan |
| Israel | Y Weiss |
| Italy | P Cotogni |
| Netherlands | J Hazelzet |
| Poland | A Mikstacki |
| Serbia | M Surbatovic |
| Spain | J Rello |
| United Kingdom | C Hinds |

**Principal Investigators**

**Austria AT**

H Novak

Belgium BE

| H Spapen  P Biston  T Dugernier  P.F. Laterre  P Damas  V Collin |  |
| --- | --- |
| **Croatia HR**  M Grgic Medic  T Mahecic |  |
| **Czech Republic CZ**  V Sramek  J Mannova  D Bares  O Marek  I Satinsky  I Novak  M Panko  S Vojtech  I Zykova |  |
| **Estonia EE**  S Sarapuu  **France FR**  J D Chiche  J L Diehl  A Rabbat  B Misset  P Asfar  H Hyvernat  P Sanchez  J-P Bedos  **Germany DE**  F Isgro  M Grundling  U Jaschinski  M Ragaller  F Bloos  S Schroder  J Krassler  A Nierhaus  C Putensen  M Weiss  Prof Larsen  M Lauterbach  **Greece GR**  D Evrenoglou  A Armaganidis  **Hungary HU**  K Darvas  I Okros  **Ireland IE**  T Ryan  M Donnelly  J Laffey  C Cody  C Motherway  D Breen  **Israel IL**  R Pizov  A Lev  Y Weiss  **Italy IT**  V. M Ranieri  S Livigni  P Pelaia  R Tufano  A.R De Gaudio  L Gattinoni  A Pesenti  M Capuzzo  G Sangiorgi  F Turani  F Conforto  F Bilotta |  |

**Netherlands NL**

B Van Der Hoven

Poland PL

| A Siemiatkowski |
| --- |
| D Maciejewski |
| M Wujtewicz |
| E Karpel |
| A Ziajka |
| R Gajdosz |
| W Gaszynski |
| A Nestorowic |
| W Kowalski |
| A Mikstacki |
| L Drobnik |
| L Krawczyk |
| J Jastrzebski |
| A Kanski |
| W Koscielniak |
| M Mikaszweska-Sokolewicz |
| K Kusza |
| A Kubler |
| B Jozef |

Serbia RS

| M Surbatovic |
| --- |
| J Jevdjic |

Spain ES

| X L Perez-Fernandez |
| --- |
| R l Sierra |
| J Sole-Violan |
| N Carrasco |
| A Margarit-Ribas |
| J C Yebenes |
| A Valverde-Conde |
| G Sirgo |
| E Gomez-Martinez |
| F F Dorado |
| L Labarta |
| L Cambra |
| M A Vidarte-Ortiz |
| M B Castello |
| J L Fernandez |
| J Gil Cebrian |
| J M Sirvent |
| M C Martin |

United Kingdom UK

| C Hinds  C Garrard  A Johnston  D Watson  S Baudouin  M Watters  R Venn  J Bion  D Higgins  M J. Garfield  S Pambakian  J Thompson  J Durcan  A Kapila  G Bellingan  S Fletcher  A Bentley  A Mallick  R Bailie  I Krupe  M Oram  M Hayes  E Wheatley  S Murdoch  S Bonner  N Webster  G Findlay  M Blunt  G Mills  G Thomas  S Drage  A Timmins  S Pesian  A Gordon  M Kuper  P Hall  P Venkatesh  J Moreno Cuesta  S Laha  A Guleri  I Smith  A Krige  P Watt |
| --- |

| **Research Nurses/Fellows** |
| --- |
| E Svoren  A Purdy  E McLees  P Hutton  P Parsons  A Smith  R Farras-Arraya  C Higham  C Ryan  C Pirie  K Mayell  K Challis  S Morris  N Waterhouse  V Flitchett  J Margalef  Dr Mowatt  P Hudson  R Gupta  J Wilde  S Lees  A Nillson  S Andrews  E Simpson  S Mappleback  S Burfield  L Sherrard Smith  V Jamieson  K Williamson  E Thomson  S Rogers  N Wilson  S Bowrey  N Rich  N Griffin-Teal  C Mitchell-Inwang  S Williams  K Swan  S Smolen  C Jones  H Prowse  N Jacques  J Atkinson  S Boluda  A Bakarr Karim  J Hyun Ryu  J Nagle  G Bercades  M Rosbergen  G Glister  F Jefferies  D Downs  K Millward  S Elliot  J Thornton  D Mawer  J Calderwood  I Whitehead  V Goodridge  K Hugill  K Colling  S Roughton  H Tennant  J Taylor  S Hall  J Addison  L Macchiovello  E Hutcheon  C Underwood  K Wong  J Collins  N Mills  E Calton  J Sorrell  S Lowes  L Ortiz-Ruiz De Gordoa  A Ghosh  O Thunder  N Wheatley  M Templeton  R Wilson  C Gibbs  L Mountford  J Gonzalez-Moreno  M Ainsworth  S Pahary  S Musaad  J Hewlett  J England  G Ward  S Nyabadza  S Clay  C Gibson  E Archer  K Hotchkiss  D Gocher  J Daglish  M Dlamini  J Baldwin  N Doherty  J Cocker  N Waddington  N Smith  D Harrison  M Bland  L Bullock  P Raymode  G Sirgo  T Lisboa  E Diaz |

**Gains study**

**List of Contributing Centres**

Broomfield Hospital, Chelmsford, UK

Addenbrookes Hospital, Cambridge, UK

Charing Cross Hospital, London, UK

Chelsea & Westminster Hospital, London, UK

Colchester General Hospital, Colchester, UK

Hammersmith Hospital, London, UK

Homerton University Hospital, London, UK

Ipswich Hospital, Ipswich, UK

Queen Elizabeth Hospital, Kings Lynne, UK

University Hospital of Lewisham, London, UK

North Middlesex Hospital, London, UK

Norfolk & Norwich University Hospital, Norwich, UK

Southend University Hospital NHS Foundation Trust, Westcliff-on-Sea, UK

St Bartholomew's Hospital (Barts), London, UK

University College Hospital, London, UK

Whittington Hospital, London, UK

St Marys Hospital, London, UK

University Hospital Coventry, Coventry, UK

Leicester General Hospital, Leicester, UK

Leicester Royal Infirmary, Leicester, UK

Queen Elizabeth Hospital Birmingham, Birmingham, UK

Wythenshawe Hospital, Manchester, UK

Royal Hallamshire Hospital, Sheffield, UK

Northern General Hospital, Sheffield, UK

Hope Hospital, Manchester, UK

Manor Hospital, Walsall, UK

Glenfield General Hospital, Glenfield, UK

Aberdeen Royal Infirmary, Aberdeen, UK

Antrim Area Hospital, Antrim, UK

Royal Blackburn Hospital, Blackburn, UK

Blackpool Victoria Hospital, Blackpool, UK

Calderdale Royal Hospital, Calderdale, UK

Huddersfield Royal Infirmary, Huddersfield, UK

Castle Hill Hospital, Hull, UK

Hull Royal Infirmary, Hull, UK

Leeds General Hospital, Leeds, UK

St James Hospital, Leeds, UK

James Cook University Hospital, Middlesbrough, UK

Royal Victoria Infirmary, Newcastle, UK

Freeman Hospital, Newcastle, UK

Royal Preston Hospital, Preston, UK

Royal Sussex County Hospital, Brighton, UK

Southmead Hospital, Bristol, UK

Frenchay Hospital, Bristol, UK

Cardiff University Hospital, Cardiff, UK

Cheltenham General Hospital, Cheltenham, UK

Frimley Park Hospital, Frimley, UK

Kettering General Hospital, Kettering, UK

John Radcliffe Hospital, Oxford, UK

Royal Berkshire Hospital, Reading, UK

Great Western Hospital, Swindon, UK

Worthing General Hospital, Worthing, UK

Southlands Hospital, Shoreham-by-Sea, UK

**GAinS study**

**Chief Investigators**

| Charles Hinds |
| --- |
| Christopher Garrard |

Principal Investigators

| Charles Hinds   |  | | --- | | Christopher Garrard | | Andrew Johnston | | Dave Watson | | Simon Baudouin | | Malcolm Watters | | Richard Venn | | Julian Bion | | David Higgins | | Mark J. Garfield | | Samuel Pambakian | | Jonathan Thompson | | John Durcan | | Atul Kapila | | Geoff Bellingan | | Simon Fletcher | | Andrew Bentley | | Abhiram Mallick | | Ingrid Krupe | | Matt Oram | | Michelle Hayes | | Elizabeth Wheatley | | Stuart Murdoch | | Stephen Bonner | | Nigel Webster | | George Findlay | | Mark Blunt | | Gary Mills | | Gareth Thomas | | Stephen Drage | | Andrew Timmins | | Siamek Pesian | | Anthony Gordon | |  | |
| --- | --- | --- | --- | --- | --- | --- | --- | --- | --- | --- | --- | --- | --- | --- | --- | --- | --- | --- | --- | --- | --- | --- | --- | --- | --- | --- | --- | --- | --- | --- | --- | --- | --- | --- |
| Research Nurses and Fellows |
| | Dr Eduardo Svoren | | --- | | Alice Purdy | | Eleanor McLees  Carmen Correia  Ying Hu  Phoebe Bodger | | Paula Hutton | | Penelope Parsons | | Alexandra Smith | | Roser Farras-Arraya | | Charley Higham | | Charlotte Ryan | | Catherine Pirie  Verity Calder  Helen Walsh  Sarah Nutbrown  Heather Payne | | Karen Mayell | | Karen Challis | | Sarah Morris  Paul Liddiard | | Nicky Waterhouse | | Valerie Flitchett  Jordi Margalef | | Dr Chris Mowatt | | Paul Hudson | | Ritu Gupta | | Jude Wilde | | Sarah Lees | | Annette Nillson  Colin Bergin  Lauren Day-Cooper  Aisling Clarkson  Joanne Millar  Annette Nilsson  Elsa Jane Perry | | Sarah Andrews | | Dr Emily Simpson | | Sarah Mappleback | | Sharon Burfield | | Loida Sherrard Smith | | Verona Jamieson | | Kim Williamson | | Emily Thomson | | Sarah Rogers | | Nicola Wilson | | Sarah Bowrey | | Natalie Rich | | Nicola Griffin  Prem Andreou  Dawn Hales  Sandra Kazembe | | Christine Mitchell-Inwang | | Sarah Williams | | Karen Swan | | Susan Smolen  Fiona McNeela | | Carys Jones | | Heather Prowse | | Nicola Jacques  Abby Brown | | Susana Boluda | | Abu-bakarr Karim | | Jung Hyun Ryu  Georgia Bercades | | Melissa Rosbergen  Georgina Glister | | Fiona Jefferies | | David Downs | | Karen Millward  Katie Mccalman  Fiona Jefferies | | Stuart Elliot  Zoe Beardow | | Judith Thornton | | Dr Damian Mawer | | James Calderwood | | Dr Iain Whitehead | | Victoria Goodridge | | Keith Hugill | | Kerry Colling | | Sian Roughton | | Heather Tennant | | Jane Taylor | | Sally Hall | | Jenni Addison | | Luis Macchiovello | | Elizabeth Hutcheon | | Carol Underwood | | Kathrine Wong | | Jane Collins | | Nathaniel Mills | | Emily Calton  John Humphreys  Julie Sorrell  Rachel Walker  Verena Hauer  David Kitson  Emily Errington  Adaeze Ochelli-Okpue  Mark Ainsworth | | Sarah Lowes | | Laura Ortiz-Ruiz De Gordoa | | Alison Ghosh | | Orla Thunder | | Natalie Wheatley | | Maie Templeton | | Robert Wilson | | Claire Gibbs | | Laura Mountford | | Juan Gonzalez-Moreno | |
| Sheik Pahary  Michele Bianchi  Jackie Hewlett  Geraldine Ward  Denise Gocher  Marie McCauley  Jacqui Daglish  Stacey Gibbons-Smith  Shilah Nyabadza  Steven Clay Catherine Gibson  Emily Archer  Karen Hotchkiss  Mabandla Dlmini  Jacqueline Baldwin  Angela Walsh  Nicola Doherty  Natalia Waddington  Neil Smith  Vicky Mendham  Martin Bland  Lynne Bullock  Donna Harrison  Parizade Raymode  Sally Grier |
| Elaine Hall |
